# Special Issue ‘Microbial glycobiotechnology’

**DOI:** 10.1186/s12934-022-01784-7

**Published:** 2022-04-07

**Authors:** Ashok Pandey, Vijai Kumar Gupta

**Affiliations:** 1 Centre for Innovation and Translational Research, CSIR-Indian Institute for Toxicology Research (CSIR-IITR), Lucknow, 226001 India; 2 Centre for Energy and Environmental Sustainability, Lucknow, 226029 Uttar Pradesh India; 3 Sustainability Cluster, School of Engineering, University of Petroleum and Energy Studies, Dehradun, 248007 Uttarkhand India; 4 Biorefining and Advanced Materials Research Center, SRUC, Kings Buildings, West Mains Road, Edinburgh, EH9 3JG UK; 5 Center for Safe and Improved Food, SRUC, Kings Buildings, West Mains Road, Edinburgh, EH9 3JG UK

## Editorial

Research and technologies involving carbohydrates to carbohydrate-based materials and converting carbohydrates for renewable energy, agri and health fall under the umbrella of glycobiotechnology. Microbial glycobiotechnology refers to sustainable bioprocesses for the bioconversion of carbohydrates through microbial enzymes and microbial technologies and the generation of tailor-made carbohydrate bioproducts/polymers for various novel applications in bioenergy and bioenergy fuels, biomaterials, in food and nutrition, and pharmaceuticals.This special issue is intended to present a special collection with a thematic scheme: Microbial Glycobiotechnology. Eighteen articles, connected to glycan biosynthesis, glyco-enzymology, modification of complex carbohydrates and their corresponding glycoconjugates, including glycans, glycoproteins, glycolipids, biological functions of glycoconjugates and glycolipids, have been published there [1–18].

## Food and nutrition

Poultry is one of the most popular animal-based food at global level, therefore, there is need to develop low-cost vaccines to protect poultry against various types of microbial infections. Nowadays, Protein Glycan Coupling Technology (PGCT) is used for development of multivalent poultry vaccine to protect against various bacterial infections.

Some researchers studied a candidate glycan-based multivalent live vaccine that elicits an immune response and protect against avian and zoonotic pathogens like *Campylobacter jejuni*, *Clostridium perfringens* and avian pathogenic *Escherichia coli* (APEC) [1]. Similarly, Samaras et al. [9] worked on the production of glycoconjugate vaccines expressed in *Escherichia coli* using an automated platform. They developed a real-time automated platform allowing the identification of the most appropriate *E. coli* strain and genetic constructs to be applied in ongoing research.

In another study, Zhang et al. [8] discussed human milk oligosaccharides, the 3^rd^ most ample constituent of human milk after lactose and lipids. They studied the effects of human milk oligosaccharides on the infant gut microbiome alongwith the mechanism of action behind the beneficial impacts of human milk oligosaccharides.

Glucosylglycerol, a natural osmolyte from bacteria and plants, is used as a cosmetic and food-and-feed ingredient. Therefore, some researchers enzymatically synthesized glucosylglycerol using a whole cell-based catalyst [10].

## Bioenergy and bioproducts

With increasing global population and industrialization, there is accelerating energy demands which is a great concern worldwide. International researchers showed increased attention towards ecofriendly producion of renewable energy fuels from different types of biomass substrates using microbial systems. Ramamurthy et al. [12] highlighted the bacterial application to develop biofuels and bioenergy.

In another study, Thangavelu et al. [2] investigated the bioconversion potential of *Candida tropicalis* ASY2 to produce biodiesel from sago processing wastewater (SWW). They observed that the microbial lipids from *C. tropicalis* strain as alternative oil substitutes for sustainable biodiesel production.

Microalgae is also an alternative to petroleum-based fuels. Sirohi et al. [3] discussed about different omics techniques to improve the product yield by algal strain manipulation. Similarly, Chuzelet al. [4] used metagenomic screening coupled with glycoanalytics to discover enzymes. In another study, Marella et al. [5] investigated the mechanisms involved in organic carbon acquisition in diatoms and the development of sustainable diatom biorefineries to produce novel biomolecules.

Microbes offer many pathways towards bioremediation and utilization of agricultural wastes. For example, Bhatt et al. [11] addressed recent advances and applications of microbial glyco-conjugates in the biological removal of organic pollutants-contaminated environments. They found that glycoconjugates create a bridge between microbes and organics, which helps to accelerate microbial degradation of pollutants. In another study, Munirajet al. [13] attempted to explore the mechanism of humic substance (HS) formation from coir pith wastes using the enzyme tyrosinase.

## Tools for biomedical research

Carvalho et al. [6] analysed fungal cell walls using recombinant endo-β(1,3)-D-glucanases. The authors observed the effectiveness and reproducibility of 12 recombinant endo-β(1,3)-D-glucanases and reported the significance of these enzymes for various biological applications.

Recombinant therapeutics plays a important role in disease management, and yeasts are used for synthesizing pharmaceutical recombinants. Madhavanet et al. [7] reviewed the development in yeast gene-manipulation techniques for biopharma protein synthesis.

In another study, Vigasovaet et al. [14] highlighted the role of multi-pathogen infections in Alzheimer’s disease (AD). They reported that the coexistence of multiple pathogens and biofilms in AD’s etiology is helpful in its diagnosis and treatment [14].

Koirala et al. [15] investigated dextran synthesis through fermentation of brewers´ spent grain (BSG), with *Leuconostoc pseudomesenteroides* DSM20193 and *Weissella confusa* A16. Moreover, Asemoloyeet al. [16] discussed different protein engineering approaches for enhanced production of important ligninolytic enzymes.

Thakur et al. [17] reported the potential uses of rhamnolipids (RLs) in cosmetic, pharmaceutical, food and healthcare industries as potential therapeutic agents. At the same time, Mo et al. [18] studied a novel multi-stress-tolerant probiotics *Meyerozyma guilliermondii* GXDK6 with aroma-producing properties and reported its potential application in the fermentation industry.
